# Immunomodulatory Effect of Phage Depolymerase Dep_kpv74 with Therapeutic Potential Against K2-Hypervirulent *Klebsiella pneumoniae*

**DOI:** 10.3390/antibiotics14010044

**Published:** 2025-01-07

**Authors:** Nikolay V. Volozhantsev, Maria A. Makarova, Alena S. Kartseva, Marina V. Silkina, Valentina M. Krasilnikova, Egor A. Denisenko, Alexander I. Borzilov, Victoria V. Firstova

**Affiliations:** State Research Center for Applied Microbiology and Biotechnology, 142279 Obolensk, Russia; mari.makar20@gmail.com (M.A.M.); kartseva_as@mail.ru (A.S.K.); marksil@yandex.ru (M.V.S.); krasv55@mail.ru (V.M.K.); egord1988@gmail.com (E.A.D.); borzilov@obolensk.org (A.I.B.)

**Keywords:** *Klebsiella pneumoniae*, bacteriophage, polysaccharide depolymerase, lymphocyte proliferation, CD69, immunomodulation

## Abstract

**Background:** The emergence of multidrug-resistant hypervirulent *Klebsiella pneumoniae* (hvKp) has made it difficult to treat and control infections caused by this bacterium. Previously, the therapeutic effectiveness of phage-encoded depolymerase Dep_kpv74 in a mouse model of *K. pneumoniae*-induced thigh soft tissue infection was reported. In this study, the effect of Dep_kpv74 on blood parameters in mice, the proliferation and subpopulation composition of spleen lymphocytes, and the activity and stability of the enzyme at different pH and temperatures were further explored. **Results:** The stability tests showed that Dep_kpv74 remained active in the temperature range from 8 °C to 55 °C. The optimal pH value for maintaining the activity of Dep_kpv74 ranged from 5.0 to 9.0. The depolymerase was detected in the blood, spleen, and lungs of mice 10 min after intraperitoneal administration, reaching maximum activity values after 1–3 h and maintaining activity a day after administration. The introduction of Dep_kpv74 at the therapeutic dose (10 μg/mouse) or at a 10-fold higher dose did not lead to reliable changes in bloodstream cell content compared with the reference values of intact mice. The biochemical results of the studies indicated that Dep_kpv74 did not exert any toxic effects on liver and kidney functions. The results of the analysis of lymphocyte proliferative activity demonstrated that Dep_kpv74 depolymerase has a mild immunomodulatory effect. **Conclusions:** Thus, the results of this study provide one more confirmation that depolymerase Dep_kpv74 is a potential candidate for the treatment of infections caused by hvKp expressing K2 capsular polysaccharides.

## 1. Introduction

Hypervirulent *Klebsiella pneumoniae* (hvKp) has become a cause of serious healthcare-associated infections and community-acquired invasive infections, manifesting as pyogenic liver abscesses (PLAs) with frequent complications, such as endophthalmitis and meningitis [1,2]. Until recently, PLA syndrome caused by hvKp was reported primarily in Southeast Asia [1], but there is increasing evidence of the syndrome occurring in other geographic regions, including North America and Europe [3,4]. Moreover, most of the currently isolated hvKp strains exhibit multidrug resistance [5,6], which significantly complicates the treatment of the diseases they cause. This problem requires the development of alternative and additional approaches and methods to treat hvKp-induced infections.

A key virulence factor of *K. pneumoniae* is the polysaccharide capsule, which has an essential increased production in most hvKp strains. The capsule encases the entire cell surface and provides hvKp resistance to host immune defense mechanisms, suppresses early inflammatory response, participates in biofilm formation, and limits the access of antibiotics and bacteriophages [7,8,9]. Disruption of the capsular polysaccharides (CPSs) of *K. pneumoniae* could be a valuable therapeutic strategy for combating hvKp-induced infections. To overcome the CPS barrier, many bacteriophages use specific enzymes (polysaccharide depolymerases), which destroy polysaccharide compounds, thereby ensuring subsequent adsorption of the phage on the outer membrane receptors, the penetration of phage DNA, and the lysis of the bacterial cell [10,11,12]. Recently, there have been several publications on the successful application of phage depolymerases in the treatment of infections caused by clinical *K. pneumoniae* strains, including hvKp strains, which are CPS hyperproducers [13,14,15,16,17,18]. However, the therapeutic use of depolymerases is lagging due to poor knowledge regarding their mechanism of action, structure–activity relationships, pharmacokinetics, stability, and safety.

Previously, the therapeutic activity of phage-KpV74-derived depolymerase Dep_kpv74 specific to hvKp expressing K2 CPS was reported [18]. Depolymerase was identified as a specific β-glucosidase that cleaved K2 polysaccharides via a hydrolytic mechanism. It should be noted that the therapeutic effect of the Dep_kpv74 protein was greater than that of the KpV74 bacteriophage. Unlike the bacteriophage, depolymerase exerted a therapeutic effect on thigh soft-tissue infection in mice caused by both a *K. pneumoniae* strain sensitive to the phage and a strain resistant to the KpV74 phage [18]. In order to use the Dep_kpv74 protein, as well as other similar depolymerases, as a therapeutic antibacterial agent, it is essential to establish the basic safety issues of its use, investigate the risk of immunogenicity associated with higher doses, and determine the activity and stability of these enzymes under different conditions in vitro and in vivo, etc.

This study aims to further explore the effects of Dep_kpv74 protein on blood parameters in mice, the proliferation and subpopulation composition of spleen lymphocytes, and the activity and stability of Dep_kpv74 under different pH and temperature conditions.

## 2. Results

### 2.1. Depolymerase Dep_kpv74 Activity and Stability

As shown previously, the phage-derived depolymerase Dep_kpv74 was specific to hvKp of the K2 capsular type [18]. The Dep_kpv74 protein produced a translucent spot that resembled the phage plaque halo (Figure S1) on the bacterial lawn of *K. pneumoniae* strains of the K2 capsular type, including K2 hvKp strains. Dep_kpv74 activity against *K. pneumoniae* strains of other capsule types was not detected (Table S1).

**Figure 1:**
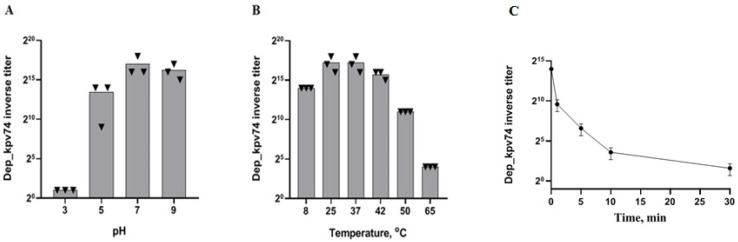
Stability assay of depolymerase Dep_kpv74. (**A**) Effect of pH on the Dep_kpv74 activity; (**B**) effect of various temperatures (pH 7.4) on the Dep_kpv74 activity; (**C**) residual activity of Dep_kpv74 after incubation at 65 °C for 30 min.

The optimal activity and stability of Dep_kpv74 with regard to pH and temperature were further determined by titration of depolymerase on the lawn of *K. pneumoniae* KPi1627 cells. Depolymerase activity was defined as the inverse titer equal to the largest serial dilution for which a detectable translucent spot on the bacterial lawn was observed. The results of the experiments showed that Dep_kpv74 retained most of its activity at pH values ranging from 5.0 to 9.0. When the cells were exposed to pH 3.0, the enzyme activity decreased significantly (Figure 1A).

In the thermal stability test, Dep_kpv74 depolymerase was relatively stable at temperatures between 8 °C and 55 °C (Figure 1B). The depolymerase activity was significantly decreased by incubation at 65 °C for 1 min. Nevertheless, residual activity was retained after 30 min of incubation at 65 °C (Figure 1C).

**Figure 2:**
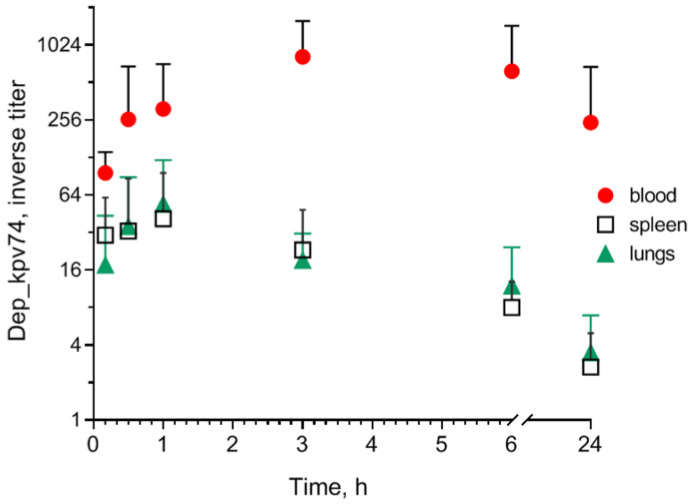
Biodistribution of Dep_kpv74 depolymerase in the blood, spleen, and lungs of outbred mice after intraperitoneal administration at a dose of 50 μg/mouse.

In an experiment on outbred mice, Dep_kpv74 depolymerase was detected in the blood, spleen, and lungs 10 min after intraperitoneal administration, reaching maximum activity values after 1–3 h and maintaining activity a day after administration (Figure 2).

### 2.2. Effect of Depolymerase Dep_kpv74 on Blood Parameters in Mice

In subsequent experiments, BALB/c mice (female) were injected once with either 10 or 100 μg of depolymerase Dep_kpv74 in 0.5 mL saline solution. Mice in the control group were injected with saline alone. After depolymerase administration, all mice were healthy throughout the observation period, without any abnormal behavior or obvious differences in body weight gain compared with intact animals that were not injected with depolymerase.

At autopsy of euthanized mice, the morphological patterns of the spleen, liver, and lungs in the animals injected with Dep_kpv74 did not differ from those in the control mice.

**Figure 3:**
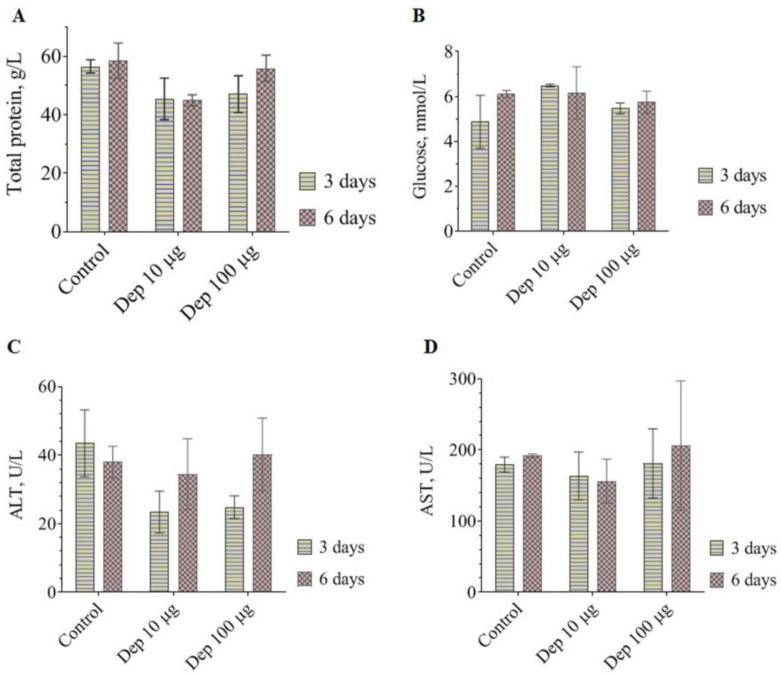
Blood biochemical parameters in female BALB/c mice on days 3 and 6 after administration of 10 μg or 100 μg of Dep_kpv74 depolymerase. Control, corresponding parameters in mice not injected with depolymerase. Total protein (**A**), glucose (**B**), ALT (**C**), and AST (**D**) values are shown.

On the 3rd and 6th days after the mice were injected with Dep_kpv74, the average contents of total blood protein and glucose, as well as the aspartate aminotransferase (AST) values, did not differ from the corresponding values in the mice of the control group (Figure 3A,B,D). On the 3rd day, a 1.7-fold decrease in alanine aminotransferase (ALT) activity was observed in the experimental group compared with the control group. The ALT level was restored on day 6 after depolymerase administration (Figure 3C).

The results of the analysis of blood hematological parameters in the intact mice and mice injected with depolymerase did not reveal significant changes in any of the indicators, even after administration of 100 μg of Dep_kpv74. No significant difference was observed in the hematological parameters of the mice between the different injection doses of Dep_kpv74 (10 or 100 µg/mouse) and post-injection time (3rd and 6th days) (Table 1).

### 2.3. Effect of Depolymerase Dep_kpv74 on the Proliferation and Subpopulation Composition of Lymphocytes

Lymphocyte proliferation is an important indicator of functional immune system activity. Spleen cells from mice injected once with 10 or 100 μg of depolymerase were used to assess Dep_kpv74-induced lymphocyte proliferation.

**Figure 4:**
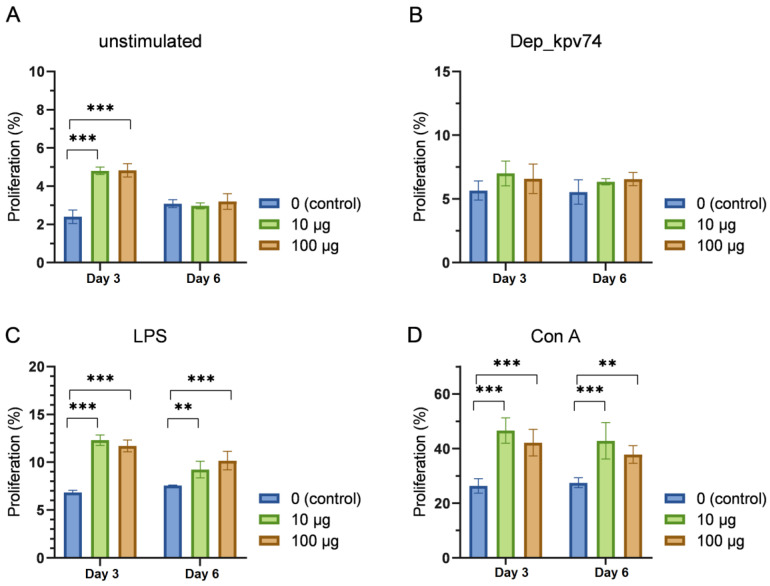
Proliferative activity of lymphocytes in BALB/c mice on 3rd and 6th days after administration of 10 or 100 µg of depolymerase Dep_kpv74. (**A**) Unstimulated lymphocytes; (**B**–**D**) in vitro stimulation of lymphocytes by depolymerase Dep_kpv74, LPS, and ConA, respectively. Blue bars, control groups of mice without depolymerase injected; green bars, mice injected with 10 μg of depolymerase; brown bars, mice injected with 100 μg of depolymerase. *n* = 5 per group, means ± SD are plotted; ** *p* < 0.001, *** *p* < 0.0001 according to two-way ANOVA followed by Tukey’s multiple comparisons test.

The results of the analysis of lymphocyte proliferative activity demonstrated an increase in spontaneous lymphocyte proliferation on the 3rd day following the injection of depolymerase Dep_kpv74, which was normalized by the 6th day (Figure 4A). No statistically significant increase in lymphocyte proliferation was observed on either the 3rd or 6th day following the injection of depolymerase Dep_kpv74 when lymphocytes were restimulated in vitro by this protein (Figure 4B). The stimulation of lymphocytes by LPS, which is known to primarily activate B lymphocytes, resulted in enhanced lymphocyte proliferation in the Dep_kpv74-injected mice (Figure 4C). In addition, an increase in lymphocyte proliferation was observed on both the 3rd and 6th days following depolymerase injection when lymphocytes were stimulated in vitro by concanavalin A (ConA), a T lymphocyte activator (Figure 4D). It should be noted that all the mentioned changes were equally observed in the mice that were administered both 10 μg and 100 μg of depolymerase. No statistically significant differences in lymphocyte proliferation were observed among the mice injected with different depolymerase doses (10 or 100 μg). Representative flow plots showing the frequency of proliferating lymphocytes in the mice injected with 10 μg of Dep_kpv74 are demonstrated in Figure S2.

### 2.4. Phenotyping of Splenocytes from Mice Injected with Depolymerase Dep_kpv74

**Figure 5:**
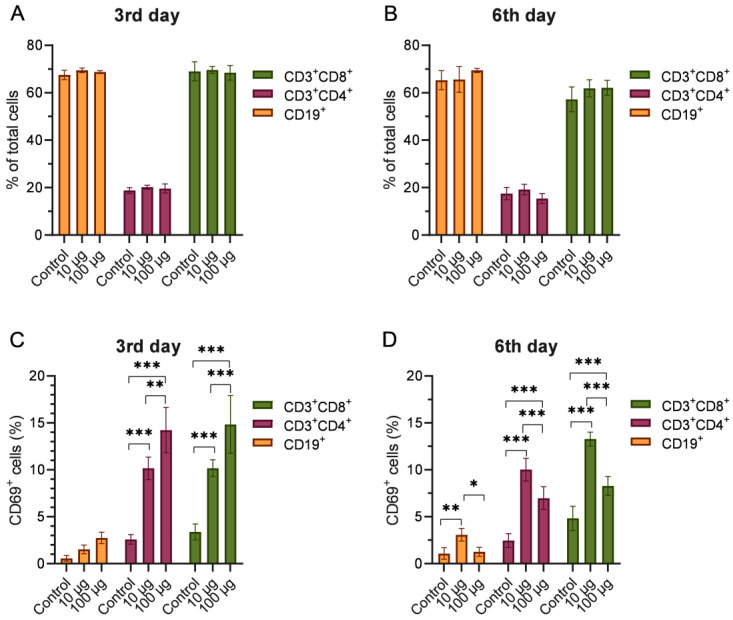
Percentage of T helper cells (CD3^+^CD4^+^), cytotoxic T cells (CD3^+^CD8^+^), and B cells (CD19^+^) in the mouse spleens on the 3rd (**A**) and 6th (**B**) days after administration of Dep_kpv74. Comparisons of the expression of CD69 in different cell subsets on the 3rd (**C**) and 6th (**D**) days after administration of Dep_kpv74. *n* = 5 per group, means ± SD are plotted; * *p* < 0.01, ** *p* < 0.001, *** *p* < 0.0001 according to two-way ANOVA followed by Tukey’s multiple comparisons test. Representative flow cytometry plots are presented in Figure S3.

As demonstrated by flow cytometry analysis, there were no statistically significant differences in the percentage of subpopulations of T helper cells (CD3^+^CD4^+^), cytotoxic T cells (CD3^+^CD8^+^), and B lymphocytes (CD19^+^) between the mice injected with Dep_kpv74 and the intact mice (Figure 5A,B).

CD69 is a surrogate marker of T cell responsiveness to mitogen and antigen stimuli and can be used as a measure of T lymphocyte activation. In this case, the results of the flow cytometry analysis showed a statistically significant increase in the number of activated T helper (CD3^+^CD4^+^CD69^+^) and activated cytotoxic lymphocyte (CD3^+^CD8^+^CD69^+^) subpopulations in mice on day 3 after administration of both depolymerase doses compared with the intact group (Figure 5C). Furthermore, the injection of the mice with Dep_kpv74 at a dose of 100 μg resulted in a more pronounced increase in CD69^+^ T helper and cytotoxic T lymphocyte counts than that observed with a dose of 10 μg of depolymerase.

On day 6, increased numbers of activated T helper cells, cytotoxic lymphocytes, and B lymphocytes were observed following injection with a lower dose of Dep_kpv74 (10 μg/mouse) (Figure 5D). Injection of mice with a dose of 100 μg of depolymerase resulted in an increase in the number of CD69^+^ T helper cells and cytotoxic lymphocytes but not B lymphocytes.

## 3. Discussion

Phage-encoded polysaccharide depolymerases have shown great antibacterial potential for treating bacterial infections in animal disease models [13,14,15,16,17,18,19]. However, in order to use depolymerase as a therapeutic antibacterial agent, it is essential to establish the basic safety issues of its use, investigate the risk of immunogenicity associated with higher doses, and determine the activity and stability of these enzymes under different conditions in vitro and in vivo, etc.

In this study, the effects of *K. pneumoniae*-type K2-specific depolymerase Dep_kpv74 on blood parameters in mice and the proliferation and subpopulation composition of spleen lymphocytes were investigated. The enzyme activity and stability were also evaluated at various pH values and temperatures.

The results indicated that the optimal pH value for maintaining the activity of Dep_kpv74 ranged from 5.0 to 9.0, whereas a solution pH of 3.0 resulted in a substantial decrease in depolymerase activity. These data are consistent with the results of other studies examining the pH stability of depolymerases of *K. pneumoniae* phages. Majkowska-Skrobek et al. [15] reported that depolymerases KP32gp37 and KP32gp38 specific for *K pneumoniae* CPS of K3 and K21 types, respectively, showed optimal stability under slightly alkaline conditions (pH 7.0–9.0). Under acidic conditions (pH 6.0 and below), less than 30% of the original activity of these enzymes was preserved. Optimal depolymerase stability under slightly alkaline conditions was also demonstrated by Bessler et al. [20] and Wu et al. [21]. On the contrary, depolymerase depoKP36 from the bacteriophage KP32 specific to *K. pneumoniae* CPS K63 type was found to be stable in a pH range shifted toward more acidic conditions, from 4.0 to 7.0 [14].

Temperature stability in the range of 18 °C–45 °C was also observed for the depolymerases KP32gp37 and KP32gp38 [15]. The Dep_kpv74 remained stable in the temperature range from 8 °C to 55 °C. Incubation at 65 °C for 1 min significantly reduced the activity of the enzyme. Such thermal stability is relatively low compared to other Klebsiella phage depolymerases. For example, Wu et al. [21] reported that the recombinant depolymerase Dep42 from the K47-specific phage SH-KP152226 was highly active up to 80 °C.

Previously, the therapeutic effectiveness of Dep_kpv74 depolymerase was demonstrated in a mouse model of thigh soft tissue with *K. pneumoniae* infection. A single administration of depolymerase at a dose of 10 μg/mouse ensured the survival of 80% of animals infected with a lethal dose of the hypermucoid hvKp strain [18]. In this study, to identify the immunogenic properties of Dep_kpv74, mice were injected with depolymerase at a therapeutic dose (10 μg/mouse) or at a 10-fold higher dose (100 μg/mouse).

The introduction of Dep_kpv74 at doses of 10 or 100 μg/mouse did not lead to reliable changes in the bloodstream cell content compared with the reference values of intact mice (control group) on the 3rd and 6th days of the study.

The gold standard for monitoring hepatotoxicity is the activity of serum liver transaminases (ALT and AST). In our study, the introduction of the depolymerase did not lead to reliable changes in AST levels in mouse blood serum (Figure 3D). A decrease in ALT was observed on the 3rd day after the introduction of Dep_kpv74, and the level was restored by the 6th day (Figure 3C). High ALT levels are a specific biomarker of hepatotoxicity, whereas low ALT levels are generally considered good and usually not a cause for concern [22].

The functional activity of lymphocytes in the mice on the 3rd day following the injection of Dep_kpv74 demonstrated an increase in spontaneous lymphocyte proliferation (Figure 4A). In addition, ConA- or LPS-stimulated proliferation of lymphocytes was enhanced in the mice injected with Dep_kpv74 compared with that in the intact mice (Figure 4C,D). Lymphocyte proliferation activity was not increased in lymphocytes from either the Dep_kpv74-injected or intact mice when stimulated in vitro with depolymerase (Figure 4B). Concanavalin A is an antigen-independent mitogen and functions as a signal one inducer, leading T cells to polyclonal proliferation [23]. LPS stimulates the proliferation of polyclonal mouse B cells and is considered a mitogen for these cells [24]. Our data provide compelling evidence for the existence of the immunomodulatory capacity of Dep_kpv74, but no specific proliferation to Dep_kpv74 was found.

CD69 is a type II integral membrane protein with a C-type lectin domain, usually identified as a classical early marker of lymphocyte activation [25]. Injection of the mice with Dep_kpv74 resulted in an increase in the number of CD69^+^ lymphocytes on days 3 and 6 post administration. In the Dep_kpv74-injected mice, there was a more pronounced increase in both the number of activated T helper (CD3^+^CD4^+^CD69^+^) and cytotoxic (CD3^+^CD8^+^CD69^+^) lymphocyte subpopulations compared with the number of activated B cells (CD19^+^CD69^+^). At the same time, there were no statistically significant differences in the percentage of subpopulations of T helper cells (CD3^+^CD4^+^), cytotoxic T cells (CD3^+^CD8^+^, and B-lymphocytes (CD19^+^) between the mice injected with Dep_kpv74 and intact mice. Further studies are required to assess the duration of the presence of activated T and B lymphocytes in the spleen after Dep_kpv74 administration.

In conclusion, phage depolymerases represent a potential alternative strategy for controlling infections mediated by *K. pneumoniae* expressing CPS, including hvKp strains. However, evidence of the safety of this type of therapy and a comprehensive understanding of the interaction between depolymerase and the human host are needed. The presented studies showed that Dep_kpv74 depolymerase, which is active against *K. pneumoniae* of a K2 capsular type, has an immunomodulatory effect, but it has no or only a small effect on the changes in hematological, immunological, and biochemical parameters.

## 4. Materials and Methods

### 4.1. Bacterial Strains

The *K. pneumoniae* strains of different capsular types (Table S1) used in this study were obtained from the State Collection of Pathogenic Microorganisms and Cell Cultures (Obolensk, Russia). Nutrient Medium No. 1 (Obolensk, Russia), Luria–Bertani (LB) broth (Difco, Atlanta, GA, USA), or LB agar were used for bacterial strain cultivation. The string test [1] was used for detection of *K. pneumoniae* hypermucoviscosity. The strain KPi1627 (SCPM-O-B-7849; GenBank: JADOFI000000000.1) of *K. pneumoniae* capsular type K2 was used as a reference for depolymerase testing.

### 4.2. Recombinant CPS Depolymerase Preparation

*E. coli* BL21 (DE3) containing the plasmid pET22b-kpv74_56 expressing Dep_kpv74 depolymerase with the His-tag (attached to the C-terminal end) were grown in LB supplemented with 100 μg/mL ampicillin at 37 °C and at 160 rpm to OD_600_ = 0.5. Then, isopropyl-β-d-thiogalactopyranoside was added to 0.2 mM, and incubation was continued at 20 °C and at 160 rpm for 20 h. The culture fluid was centrifuged at 4 °C and 6000× *g* for 20 min, and the cell pellet was resuspended in phosphate-buffered saline (PBS) and centrifuged under the same conditions. The cell pellet was resuspended in His-tag column binding buffer (20 mM Tris–HCl, pH 7.2, 50 mM NaCl, 20 mM imidazole). Lysozyme was added to 100 μg/mL, and the mixture was incubated on ice for 15 min. Triton X-100 was added to a final concentration of 0.5%, and incubation was continued on ice for 10 min. Then, MgCl (final concentration of 1 mM) and DNase (final concentration of 10 μg/mL) were added, and the mixture was incubated at room temperature for 10 min. The lysate was clarified by centrifugation at 11,000× *g* for 20 min at 4 °C. The supernatant was loaded onto nickel Ni^2+^-charged 5 mL GE HisTrap columns (GE Healthcare Life Sciences, Marlborough, MA, USA), equilibrated with His-tag column binding buffer, and eluted with buffer containing 20 mM Tris–HCl, 50 mM NaCl, and 500 mM imidazole at pH 7.4. The fractions containing the target protein were pooled and then dialyzed against two changes of 0.01 M PBS, pH 7.4, lyophilized, and stored at −20 °C. For further experiments, depolymerase was dissolved in PBS.

Before the animal experiments, the safety of the isolated depolymerase was confirmed in outbred mice (*n* = 5) by injecting them with Dep_kpv74 at a concentration of 250 μg/mouse. Within 5 days after injection, all mice were healthy, without any abnormal behavior or obvious differences in body weight gain compared with intact animals.

### 4.3. Determination of Dep_kpv74 Activity and Stability

The Dep_kpv74 activity was determined in spot test using *K. pneumoniae* strains of different capsular types [26].

To estimate the pH-dependent stability, Dep_kpv74 was dissolved in different buffers with pH 3.24, 5.22, 7.00, and 9.21 and incubated for 10 min at 37 °C. To assess thermostability, the depolymerase was incubated in 50 mM NaH_2_PO_4_-Na_2_HPO_4_ buffer (pH 7.4) at temperatures ranging from 8 °C to 65 °C for 10 min. To determine the activity of the depolymerase after different treatments, serial 2-fold dilutions of the depolymerase solutions were inoculated on the lawn of *K. pneumoniae* KPi1627 cells, and the maximal dilution that formed a translucent halo on the bacterial lawn was determined. Depolymerase activity was defined as the inverse titer equal to the largest serial dilution for which a detectable translucent spot on the bacterial lawn was observed. All experiments were performed in triplicate.

### 4.4. Determination of Depolymerase in Mouse Blood and Organs

Outbred mice (18–20 g) were used in the experiments. Mice were housed under standard conditions of light, temperature, and water and food availability.

Mice were injected intraperitoneally with a depolymerase solution, 50 μg/mouse in PBS, and randomly divided into 6 groups, 5 in each. After 10 and 30 min and 1, 3, 6, and 24 h, the mice of the corresponding groups were euthanized, and their blood, spleen, and lungs were collected. Blood serum, spleen, and lung suspensions were titrated, and serial 2-fold dilutions were inoculated on *K. pneumoniae* KPi1627 lawns. The presence of translucent spots at the sample application site indicated depolymerase activity. Blood serum and organ suspensions from intact mice not injected with depolymerase were used as controls. Comparative depolymerase activity was determined as the reverse titer of the highest serial dilution at which a translucent spot was observed on the bacterial lawn.

### 4.5. Blood Parameter Tests

Female BALB/c mice were divided into 3 groups (10 in each group) and intraperitoneally injected with a depolymerase solution at a dose of 10 or 100 μg/mouse in PBS (2 experimental groups) or with saline alone (control group). Half of the mice from each group were euthanized after 3 and 6 days, respectively, and their blood and spleens were collected.

Whole blood was used to determine hematological parameters, and blood serum was used to determine blood biochemical parameters. The analysis of the parameters was performed using a PCE90Vet automated analyzer (High Technology Inc., North Attleboro, MA, USA). Splenocytes were used to determine the proliferative activity of lymphocytes and phenotyping.

### 4.6. Preparation and Stimulation of Splenocytes

A suspension of splenocytes was prepared under sterile conditions by homogenizing mouse spleens through a 70 μm cell strainer (Corning, Glendale, AZ, USA) in 3 mL of RPMI-1640 medium (Sigma-Aldrich, Burlington, MA, USA ) supplemented with 10% fetal bovine serum (HyClone, Northumberland, UK). Cells were precipitated by centrifugation at 200× *g* for 10 min. The number and viability of cells were evaluated using the automatic counter “TC-20” (Bio-Rad, Hercules, CA, USA) after staining with trypan blue (Invitrogen, Carlsbad, CA, USA). A total of 1 × 10^6^ cells/well were seeded in a round-bottom 96-well plate. Cells were stimulated with the recombinant Dep_kpv74 protein (10 μg/mL) as a specific antigen or with ConA (Sigma-Aldrich, Burlington, MA, USA) at 5 μg/mL or LPS *E. coli* (10 μg/mL, Sigma-Aldrich, USA) as a mitogen. As a control, spleen cells left without any activation were used.

### 4.7. Lymphocyte Proliferation Assay

To assess the proliferative activity of lymphocytes, cells were pre-stained with CFSE fluorescent dye according to the manufacturer’s instructions (Invitrogen, USA).

Following a 3-day incubation period with ConA and a 5-day incubation period with LPS or Dep_kpv74, the number of proliferating cells was determined via flow cytometry, with the level of CFSE fluorescence serving as the indicator. Finally, the samples were resuspended in 150 μL of 4% formaldehyde solution and analyzed using a BD FACSAria III flow cytometer with the FACSDiva 8.0 software.

### 4.8. Flow Cytometry Analysis of Surface Markers

Cells were collected and stained with conjugated monoclonal antibodies (mAbs) against cell surface markers for 30 min at 4 °C. The following mAb conjugates were used (BD Biosciences, Franklin Lakes, NJ, USA): CD3-FITC (clone 17A2), CD4-APC (clone RM4-5), CD8-PE (clone 53-6.7), CD19-APC (clone 1D3), and CD69-BV421 (clone H1.2F3). The samples were washed twice with PBS containing 2% fetal bovine serum. Finally, the samples were resuspended in 150 μL of 4% formaldehyde solution and analyzed using a BD FACSAria III flow cytometer with the FACSDiva 8.0 software.

### 4.9. Statistical Analysis

The statistical analysis and representative graphs were performed using GraphPad Prism 8.0 (GraphPad Software Inc., San Diego, CA, USA). For comparisons between three or more groups, analysis was made by two-way ANOVA followed by Tukey’s multiple comparisons test with significance determined at *p* < 0.05.

## Figures and Tables

**Table 1:** Hematological parameters in BALB/c mice on 3rd and 6th days following a single administration of Dep_kpv74 at a dose of 10 μg or 100 μg per mouse.

Parameters (Unit)	Intact Mice	3 Days		6 Days	
		10 µg	100 µg	10 µg	100 µg
Leukocytes (×10^9^/L)	5.8 ± 2.3	5.5 ± 2.7	4.9 ± 0.8	5.9 ± 0.8	4.8 ± 1.4
Lymphocytes (×10^9^/L)	3.9 ± 1.2	3.3 ± 2.0	2.3 ± 1.0	5.2 ± 1.5	3.1 ± 0.7
Monocytes (×10^9^/L)	0.3 ± 0.1	0.2 ± 0.1	0.2 ± 0.1	0.4 ± 0.1	0.2 ± 0.1
Granulocytes (×10^9^/L)	1.7 ± 1.0	2.0 ± 0.6	2.4 ± 0.5	3.3 ± 0.7	1.5 ± 0.7
Lymphocytes (%)	68.1 ± 6.1	58.1 ± 6.5	46.4 ± 14.7	58.0 ± 11.3	64.8 ± 6.0
Monocytes (%)	3.8 ± 0.9	3.0 ± 0.2	4.0 ± 1.0	5.5 ± 2.4	4.0 ± 0.7
Granulocytes (%)	28.2 ± 5.2	38.9 ± 6.6	49.6 ± 13.9	36.5 ± 8.9	31.2 ± 5.3
RBC (×10^12^/L)	9.58 ± 0.31	9.66 ± 0.27	9.42 ± 0.57	9.17 ± 0.14	9.95 ± 0.66
Hemoglobin (g/L)	141 ± 5	138 ± 3	137 ± 9	119 ± 3	144 ± 9
Hematocrit (%)	44.9 ± 1.3	43.3 ± 0.9	43.0 ± 2.7	40.5 ± 1.5	46.3 ± 2.7
MCV (FL)	46.9 ± 1.2	44.8 ± 0.6	45.7 ± 0.1	44.2 ± 1.2	46.6 ± 1.3
MCH (pg)	14.6 ± 0.5	14.2 ± 0.3	14.5 ± 0.1	12.9 ± 0.2	14.4 ± 0.5
MCHC (g/L)	313 ± 14	318 ± 3	319 ± 2	293 ± 7	311 ± 1
Platelets (×10^9^/L)	792 ± 121	792 ± 225	816 ± 96	655 ± 87	702 ± 319

Data presented as mean ± standard deviation. RBC: red blood cells; MCV: mean corpuscular volume; MCH: mean corpuscular hemoglobin content; MCHC: mean corpuscular hemoglobin concentration.

## Data Availability

The data presented in this study are available on request from the corresponding author.

## Supplementary Materials

The following supporting information can be downloaded at: https://www.mdpi.com/article/10.3390/antibiotics14010044/s1, Figure S1: Spot tests of depolymerase Dep_kpv74 on *K. pneumoniae* KPi1627 lawn. Aliquots (10 μL) of serial two-fold dilutions of Dep_kpv74 were spotted onto a plate containing the *K. pneumoniae* strain. The numbers indicate the dilution factor. The initial depolymerase concentration was 0.7 mg/mL; Figure S2: Representative flow plots showing the frequency of proliferating lymphocytes in control mice (upper panel) and on day 3 after 10 μg Dep_kpv74 administration (lower panel). The lymphocytes were restimulated in vitro by Dep_kpv74, LPS, or concanavalin A (Con A), respectively; Figure S3: Representative flow plots showing the frequency of activated T helper cells (CD3^+^CD4^+^CD69^+^), cytotoxic T cells (CD3^+^CD8^+^CD69^+^), and B cells (CD19^+^CD69^+^) in control mice (upper panel) and on day 3 after 10 μg Dep_kpv74 administration (lower panel); Table S1: Depolymerase Dep_KpV74 activity against *Klebsiella pneumoniae* strains.
