# Supplementary material for: Immunomodulatory Effect of Phage Depolymerase Dep_kpv74 with Therapeutic Potential Against K2-Hypervirulent Klebsiella pneumoniae
Source: Antibiotics (Basel). 2025 Jan 7;14(1):44. doi: 10.3390/antibiotics14010044 (PMC11761230; doi:10.3390/antibiotics14010044)
Supplement: Supplementary file 1 [file antibiotics-14-00044-s001.zip › antibiotics-3339111-supplementary.pdf]

Supplementary Materials

Table S1. Depolymerase Dep_KpV74 activity against *Klebsiella pneumoniae* strains.

#	Strain	Hypermucoid phenotype	cps-type	Dep_kpv74 activity	#	Strain	Hypermucoid phenotype	cps-type	Dep_kpv74 activity
1	KPB2016K/17	-	K2	+	44	KPB1493-1	+	K1	-
2	KPB691/18-4	-	K2	+	45	KPB1802	+	K1	-
3	KPB790/18-1	-	K2	+	46	KPB2580	+	K1	-
4	KPB792/18	-	K2	+	47	KPB463-13	+	K1	-
5	KPB225/20	-	K2	+	48	KPB475	+	K1	-
6	KPB306/20	-	K2	+	49	KPB594	+	K1	-
7	KPB320/20	-	K2	+	50	KPi1683	+	K1	-
8	KPB702/18	-	K2	+	51	KPi261	+	K1	-
9	KPB853/18-1	-	K2	+	52	KPS73	+	K1	-
10	ATCC BAA2472	-	K2	+	53	ATCC 13883	-	K3	-
11	KP254	-	K2	+	54	KPB711	-	K10	-
12	KPB1102	-	K2	+	55	KPB944	-	K10	-
13	KPB1294	-	K2	+	56	KPB536-14	-	K10	-
14	KPB1299	-	K2	+	57	KPi2135	+	K20	-
15	KPB1956	-	K2	+	58	KPM-9	+	K20	-
16	KPB71	-	K2	+	59	KP5023	-	K22	-
17	KPB755	-	K2	+	60	KP5058	-	K22	-
18	KPi1582	-	K2	+	61	KPB2304-15	-	K23	-
19	KPi2807	-	K2	+	62	KPB536/17-2	-	K23	-
20	KPi3310	-	K2	+	63	KPi4275	-	K23	-
21	KPi3330	-	K2	+	64	KPB1674	-	K24	-
22	KPi4341	-	K2	+	65	KPB1125	-	K27	-
23	B 1040/18-1	-	K2	+	66	KPB941	-	K27	-
24	B 1154/18	-	K2	+	67	KPB591	-	K28	-
25	B 3002K/17	-	K2	+	68	KPB1128-1	-	K31	-
26	B 3060K/17	-	K2	+	69	KPB1434-16	-	K39	-
27	B 587/18	-	K2	+	70	KPB1667	-	K47	-
28	B 771/18	-	K2	+	71	KPB1224	-	K47	-
29	B 775/18-1	-	K2	+	72	ATCC12657	+	K54	-
30	B 784/18	-	K2	+	73	KPB690	+	K57	-
31	B 823/18-1	-	K2	+	74	KPB757	+	K57	-
32	B 2646/20	+	K2	+	75	KPB811	+	K57	-
33	ATCC43816	+	K2	+	76	KPi8289	+	K57	-
34	KPB4010	+	K2	+	77	KPB1106-2	-	K57	-
35	KPB463-16	+	K2	+	78	KPB697-1	-	K57	-
36	KPB492-16	+	K2	+	79	KPB697-2	-	K57	-
37	KPi1627	+	K2	+	80	KPB54	-	K60	-
38	KPi1748	+	K2	+	81	KPB1493-2	-	K62	-
39	KPi2965	+	K2	+	82	KPB1759	-	K62	-
40	KPi3014	+	K2	+	83	KPB420	-	K62	-
41	KPi6208	+	K2	+	84	KPB417/16	-	K64	-
42	KPB470	-	K1	-	85	KPB940	-	KL107	-
43	KPB1103	+	K1	-	86	B-3188	-	KL113	-

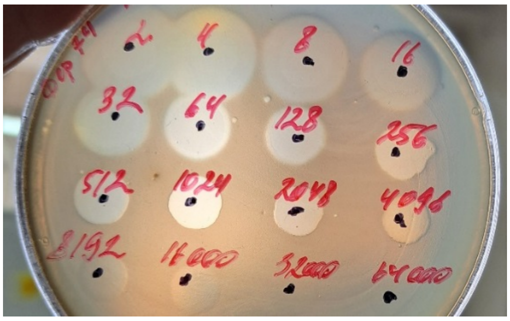

Figure S1. Spot tests of depolymerase Dep_kpv74 on *K. pneumoniae* KPi1627 lawn. Aliquots (10 µl) of serial two-fold dilutions of Dep_kpv74 were spotted onto a plate containing the *K. pneumoniae* strain. The numbers indicate the dilution factor. The initial depolymerase concentration was 0.7 mg/ml

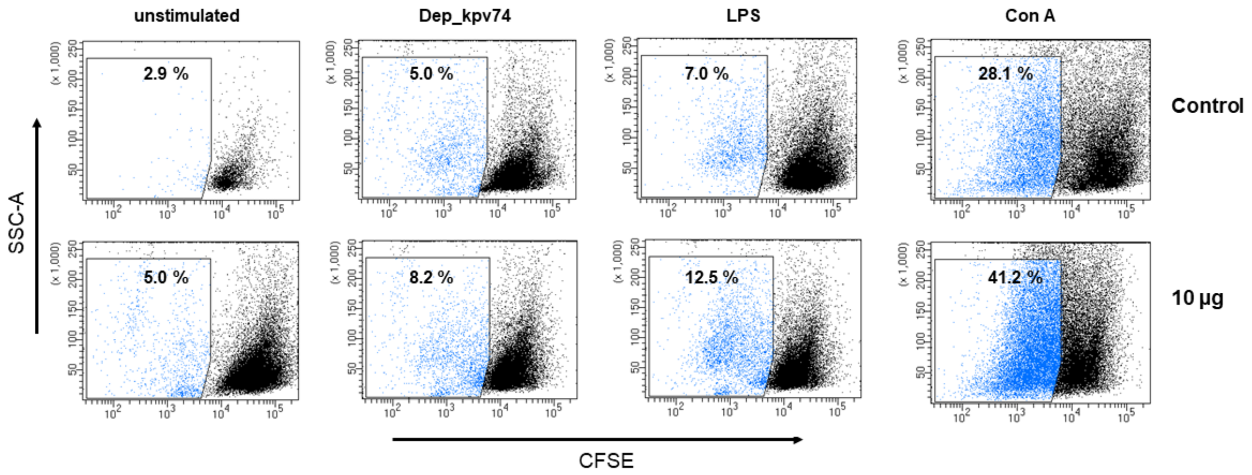

Figure S2. Representative flow plots showing the frequency of proliferating lymphocytes in control mice (upper panel) and on day 3 after 10 µg Dep_kpv74 administration (lower panel). The lymphocytes were restimulated *in vitro* by Dep_kpv74, LPS or concanavalin A (Con A), respectively.

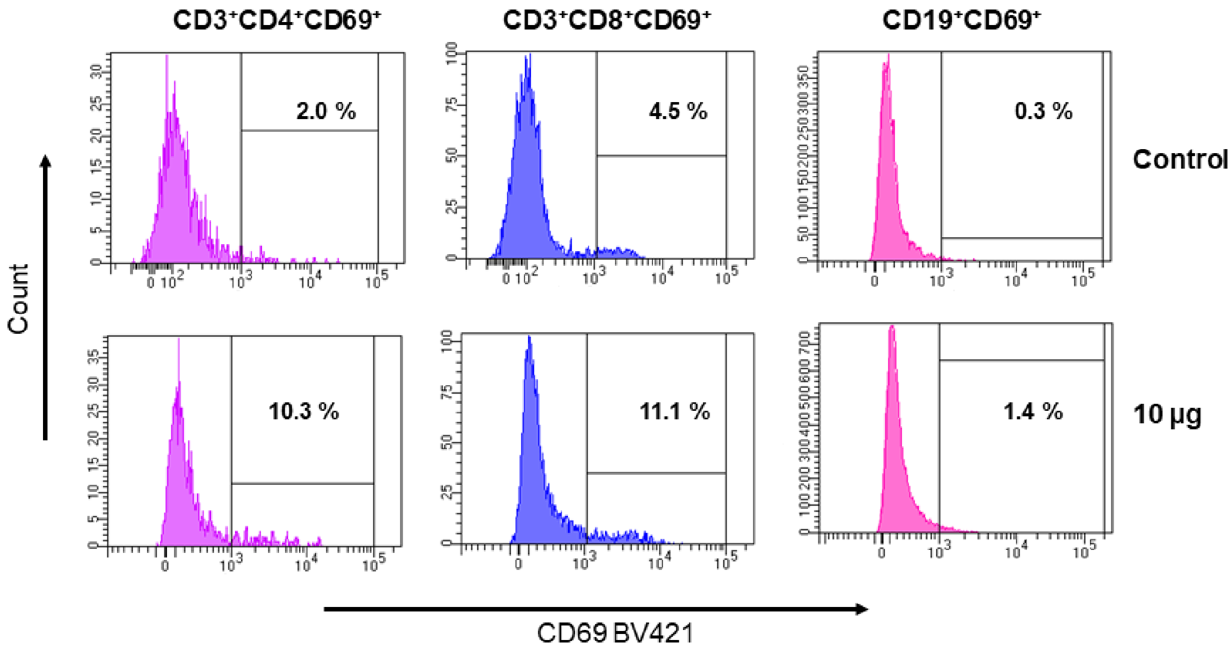

Figure S3. Representative flow plots showing the frequency of activated T helper cells ($CD3^{+}CD4^{+}CD69^{+}$), cytotoxic T cells ($CD3^{+}CD8^{+}CD69^{+}$) and B cells ($CD19^{+}CD69^{+}$) in control mice (upper panel) and on day 3 after 10 µg Dep_kpv74 administration (lower panel).
